# Effective Notch-Stress-Based Stress Concentration Factors of the Rib–Deck Weld in Orthotropic Steel Decks Considering the Effect of Asphalt Surfacing

**DOI:** 10.3390/ma16206760

**Published:** 2023-10-19

**Authors:** Qiudong Wang, Shanchun Shi, Yue Yao, Zhiqiang Wang, Zhongqiu Fu

**Affiliations:** 1 College of Civil Engineering, Nanjing Forestry University, Nanjing 210037, China; 15052610385@163.com (S.S.); wzq010302@163.com (Z.W.); 2 College of Construction Engineering, Jiangsu Open University, Nanjing 210000, China; jsouyaoyue@163.com; 3 College of Civil and Transportation Engineering, Hohai University, Nanjing 210098, China; fuzhongqiu@hhu.edu.cn

**Keywords:** orthotropic steel deck, rib–deck weld, effective notch stress, concentration factor, predictive formula

## Abstract

Effective notch stress (ENS) approaches have many application prospects in fatigue damage assessments; however, an ENS can only be obtained by conducting complex and time-consuming numerical analyses, deterring many engineers from applying such an approach. In terms of the rib–deck weld in orthotropic steel decks (OSDs), predictive formulae for determining the ENS concentration factors (ENS-based SCFs) have been proposed; however, the effect of asphalt surfacing is not involved, which limits their applications in practical engineering. In the present study, refined finite element (FE) models, including asphalt surfacing, were developed to obtain the ENS-based SCFs which could be applied to practical engineering. Parametric analyses were conducted to investigate the effect of the transverse loading position, the combined effect of the transverse loading position and asphalt surfacing, and the effect of the temperature of the asphalt surfacing. The amplification coefficients (*k_SCF_*, *k_SCF_*_1_, and *k_SCF_*_2_) were introduced to determine the ENS-based SCFs on the basis of the predictive formulae without considering the effect of asphalt surfacing. Results show that the ENS-based SCFs of the rib–deck weld is considerably affected by the transverse position of wheel loading and the asphalt surfacing. The cubic polynomial function could be employed to fit the numerical results of the ENS-based SCFs and amplification coefficients (*k_SCF_*, *k_SCF_*_1_, and *k_SCF_*_2_) with high fitting precision. Predictive formulae for determining the ENS-based SCFs corresponding to arbitrary transverse loading position and temperature of asphalt surfacing are proposed. The validation investigation turns out that the relative error of the proposed formulae is within 10%, indicating the feasibility of using this approach for engineering applications.

## 1. Introduction

Orthotropic steel decks (OSDs) are easily subjected to fatigue cracking under cyclic traffic loads [1]. As welded steel structures, there are various fatigable details in OSDs, e.g., rib–deck welds, butt welds, diaphragm–rib welds, and arc-sharped notches [2]. Among them, the rib–deck weld has generated significant concern in recent years [3]; this is because the propagation of fatigue cracks thereat will further threaten the durability of asphalt surfacing [4,5,6]. In this case, fatigue damage assessments and life predictions have become a hot issue in bridge engineering. For these purposes, several stress-based approaches have been widely employed, e.g., the nominal stress approach, the hotspot stress approach, and the effective notch stress approach [7]. The nominal stress approach is the simplest; however, the assessing accuracy is limited since the structure-related stress concentration is not considered [8,9,10]. Comparatively, the hotspot stress (HSS) approach has higher accuracy; however, such approach is limited to applications to the weld root, where most fatigue cracks of the rib–deck welds are initiated [11]. Additionally, the notch stress induced by the weld bead cannot be considered by employing the hotspot stress approach. Alternatively, the effective notch stress (ENS) approach has broad application prospects as the notch stress could be precisely considered. However, effective notch stress can only be obtained by conducting numerical simulations (e.g., finite element modeling) [12], which deters many engineers from applying such approaches in practical engineering. Exploring feasible ways to efficiently determine the effective notch stress without conducting numerical simulations is of great significance, which warrants further investigations.

Instead of complex and time-consuming finite element modeling, empirical formulae obtained using parametric and regression analysis provide a feasible way to quickly determine effective notch stress [13]. Some researchers have proposed various parametric formulae to predict the effective notch stress concentration factor (SCF), which is defined by the ratio of the effective notch stress to nominal stress. For instance, formulae for predicting the ENS-based SCFs of the butt–weld joint [13,14], the welded cruciform joint [15], and the T-butt welded joint [16,17] have been reported. ENS-based SCFs could be quickly obtained by employing these formulae for implementing accurate assessment of fatigue damage or life. In recent years, artificial neural networks (ANNs) and machine leaning have also been employed to develop accurate predictive models [18,19]. In terms of the rib–deck welded joint, Wang et al. [20] proposed predictive formulae for predicting ENS-based SCFs, corresponding to three typical fatigue-cracking modes (i.e., root–toe, root–deck, and toe–deck cracking modes) to improve the efficiency of the ENS approach. More details will be introduced in Section 2; these form the basis of the present research. Notably, the proposed formulae in [20] are only applicable and efficient when all of the geometric parameters of the rib–deck weld are accessible, while might be inapplicable due to the absence of critical geometric parameters in practical engineering, e.g., the weld leg lengths. In this case, predictive formulae for determining the ENS-based SCFs have been proposed through an integration of the HSS measurements to improve the efficiency of applying an ENS approach [21]. However, in these investigations, the effect of the asphalt surfacing is not considered, which might affect the accuracy of assessments in practical engineering applications.

This study focuses on the parametric formulae for predicting ENS-based while SCFs considering the effect of asphalt surfacing. Firstly, predictive formulae for predicting the ENS-based SCFs of a rib–deck weld, without considering the effect of the asphalt surfacing, were reviewed; these form the basis of the present study. Subsequently, a refined finite element model was developed to conduct the parametric analysis of the effect of the asphalt surfacing, wheel loading position, and temperature. Next, new predictive formulae were proposed based on the results of parametric analysis. Finally, the application of the proposed formulae was presented to validate the feasibility and accuracy.

## 2. Review of Equations for Predicting the SCF

**Figure 1:**
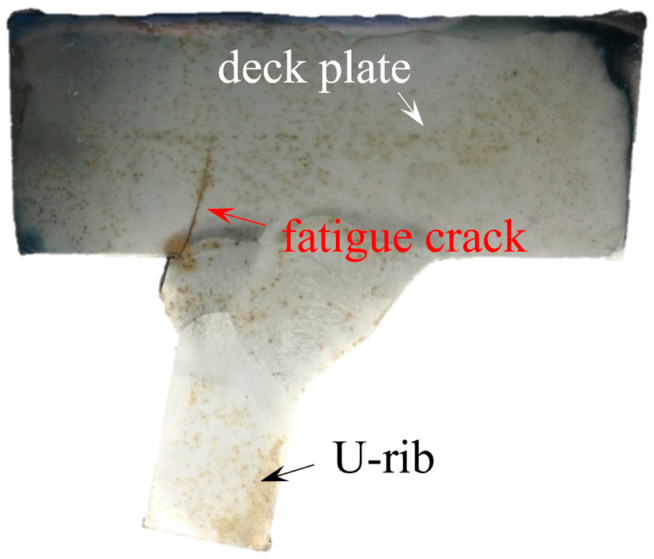
The rib–deck weld with fatigue crack penetrating the deck plate (imaged by Q.W.).

Rib–deck welds in OSDs are easily subjected to fatigue cracking, as shown in Figure 1, where the crack has penetrated the deck plate and subsequent leakage and corrosion were observed. To propose the predictive formulae for predicting the ENS-based SCFs, finite element (FE) models simulating the rib–deck welded detail were developed. For detailed geometric sizes of the FE model, please refer to [20]. Since the ENS-based SCF at a weld root was the focus of the present study, the stress 20 mm away from the weld root was selected to be the nominal stress [20]. Regarding the geometric parameters of the rib–deck weld, six parameters were considered in the FE modeling, namely the thickness of the deck plate (*t_d_*), the thickness of the rib wall (*t_r_*), the angle between the deck plate and rib wall (*θ*), the weld penetration rate (1 − *t_p_/t_r_*), the length of the weld leg at the deck plate (*l_w,d_*), and the length of the weld leg at the rib wall (*l_w,r_*). For more details of the geometric parameters, please refer to [20]. In parametric analysis, the *t_r_* was valued at 6, 8, and 10 mm, while the relative value of *t_d_*/*t_r_* was set to be 1.8, 2.0, and 2.5 to take in account the effect of the thickness of the deck plate and rib wall. The value of (1 − *t_p_/t_r_*) was increased from 0 (i.e., no penetration) to 0.8 at intervals of 0.1. The relative length of the weld leg (i.e., *l_w,d_*/*t_r_* and *l_w,r_*/*t_r_*) was valued from 0.8 to 1.2 at intervals of 0.2. Additionally, the value of *θ* increased from 70° to 80° at intervals of 5°. Based on the results of the parametric analysis, a database of the ENS-based SCFs in the presence of different geometric parameters was developed. Finally, predictive formulae for determining the ENS-based SCFs were proposed based on regression analysis, as expressed by Equation (1).

(1) $${SCF}_{ENS}=\sum_{i=1}^{28}c_{i}\times f_{i}(X_{1},X_{2},X_{3},X_{4},X_{5},X_{6})$$

where *SCF*_ENS_ represents the ENS-based SCF at the weld root; *X*_1_ = 1 − *t_p_/t_r_*; *X*_2_ = *t_r_*/10; *X*_3_ = *l_w,d_*/*t_r_*; *X*_4_ = *l_w,r_*/*t_r_*; *X*_5_ = 2*θ*/180°; and *X*_6_ = *t_d_*/*t_r_*. *c_i_* is the coefficient determined by the regression analysis as listed in [20]. Results of the parametric analysis in [20] show that the SCF is considerably affected by the weld penetration rate (*X*_1_ = 1 − *t_p_/t_r_*), the relative thickness of the deck plate (*X*_6_ = *t_d_*/*t_r_*), and the relative length of the weld leg at the rib wall (*X*_4_ = *l_w,r_*/*t_r_*). Accordingly, the SCF is less affected by the thickness of the rib wall (*X*_2_ = *t_r_*/10), the relative length of the weld leg at the deck plate (*X*_3_ = *l_w,d_*/*t_r_*), and the angle between the deck plate and rib wall (*X*_5_ = 2*θ*/180°). It is noted that the effect of asphalt surfacing on the values of *SCF*_ENS_ is not considered in Equation (1), which is to be investigated in the following sections.

## 3. Finite Element Model for Parametric Analysis

**Figure 2:**
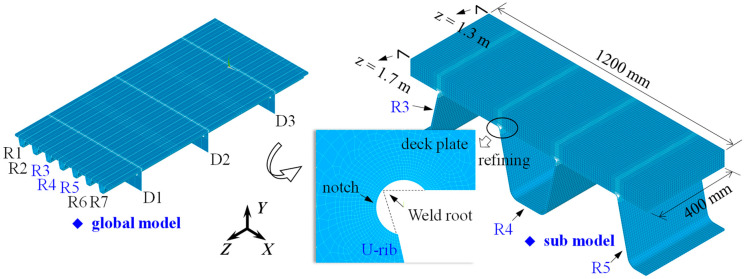
Finite element model simulating the OSD and asphalt surfacing.

**Figure 3:**
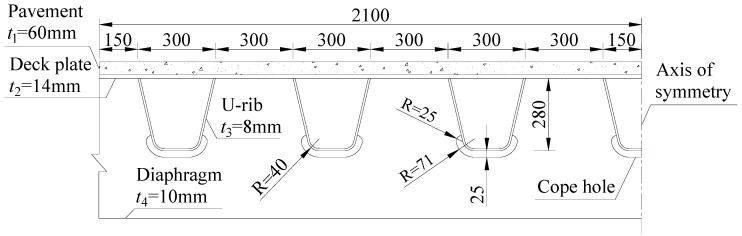
Main geometric parameters of the FE model (unit: mm).

To investigate the effect of the asphalt surfacing on the ENS-based SCFs of the rib–deck weld, refined finite element models, including the global model and sub model, were developed using ANSYS (Canonsburg, PA, USA), as shown in Figure 2. The global model was composed of seven U-ribs (labelled from R1 to R7) and three diaphragms (labelled from D1 to D3). Previous investigations have already shown that such FE model could be employed to obtain the stress characteristics of the OSDs in practical engineering with high accuracy. The thickness of the asphalt surfacing was set to be 60 mm which was in accordance with the value in extensive practical engineering. Other main geometric parameters were shown in Figure 3. The longitudinal length of the global model was 9000 mm, while the length between either two diaphragms was 3000 mm. The sub model included the U-rib labelled R4 and half of R3 and R5 (see Figure 2). The rib–deck weld of R4 was selected as the objective, and the notch with a radius of 1 mm was arranged at the weld root for obtaining the ENS, following the recommendations by the IIW [7], to account for the variation in the weld shape parameters, as well as the nonlinear material behavior at the notch root.

The SOLID186-typed element, which was defined by 20 nodes having three degrees of freedom per node, was employed to simulate both the OSD and asphalt surfacing with a meshing size of 10 mm [2]. To improve the evaluating accuracy, the mesh of the objective weld (i.e., arranged with a notch) was refined (see Figure 2), and the refined meshing size around the notch was nearly 0.2 mm [20,21]. The elastic modulus and Poisson’s ratio of the material of the OSD were 210 GPa and 0.3, respectively. As previous research has revealed, the stress characteristics of the OSD were less affected by the Poisson’s ratio of the asphalt surfacing; this was valued as 0.3 in the present study. While the elastic modulus of the asphalt surfacing was to be assigned with different values to simulate the effect of the temperature, as will be introduced in the following sections.

In terms of the boundary conditions, the displacement of nodes on the longitudinal end of the deck plate and the U-rib was constrained to simulate the continuous structure that is present in real-world engineering. The displacement of nodes on the transverse end of the deck plate and diaphragm were also constrained. The displacement of nodes at the bottom of the diaphragm was also constrained to simulate the continuous structure.

## 4. Modified Formulae for Predicting the SCFs

As illustrated before, the effect of the asphalt surfacing is not considered in the results of the ENS-based SCFs predicted by Equation (1). Therefore, parametric analysis of the effect of the asphalt surfacing, including the effect of transverse loading position and temperature, was carried out in the present study to clarify the effect of the asphalt surfacing on the ENS-based SCFs predicted by Equation (1). Notably, the effect of the transverse loading position should be taken into account because vehicles generally pass through the same cross-section following different transverse in-lane positions. As will be pointed out in Section 4.1, the ENS-based SCF determined by Equation (1) corresponds to the same transverse loading position, which cannot be directly applied to the evaluation in practical engineering as the real transverse loading position is unclear. In this case, an additional parametric analysis on the effect of the transverse loading position on the SCF should be carried out based on the FE model. Once the modified formulae were proposed, the ENS-based SCFs considering the effect of the asphalt surfacing could be efficiently obtained.

Furthermore, previous study reveals that the ENS concentration position is independent of the transverse loading position, while being sensitive to the temperature of the asphalt surfacing [2]. However, in terms of the full-penetrated rib–deck weld, the root–deck crack (i.e., the target crack in the present study) will be generated due to fatigue damage accumulation regardless of the variation in the transverse loading position and temperature of the asphalt surfacing. In the following sections, parametric analysis was performed based on the FE models with fully penetrated rib–deck welds. Therefore, the predicted ENS-based SCF could be employed to evaluate the fatigue damage in the corresponding concentration position, i.e., the proposed predictive formulae are applicable to the fatigue evaluation of the root–deck crack.

### 4.1. Effect of the Transverse Loading Position

**Figure 4:**
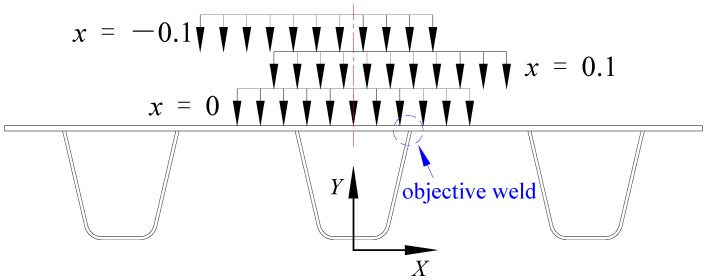
Schematic diagram of the transverse loading position (unit: m).

Firstly, to investigate the effect of single factor (i.e., the transverse loading position), an additional FE model without the asphalt surfacing was developed. The double wheel load model, with a loading area of 600 mm (transverse) × 200 mm (longitudinal), was employed according to the Chinese standard “Specifications for Design of Highway Steel Bridge” [22]. Five transverse loading positions were considered: *x* = 0, *x* = ±0.1 m, and *x* = ±0.2 m. *x* = 0 meant that the wheel loading was applied right above the U-rib labelled R4, as shown in Figure 4; *x* = 0.1 m meant that the wheel loading was 0.1 m right of the symmetric axis of the sub model; *x* = −0.2 m meant that the wheel loading was 0.2 m left.

**Figure 5:**
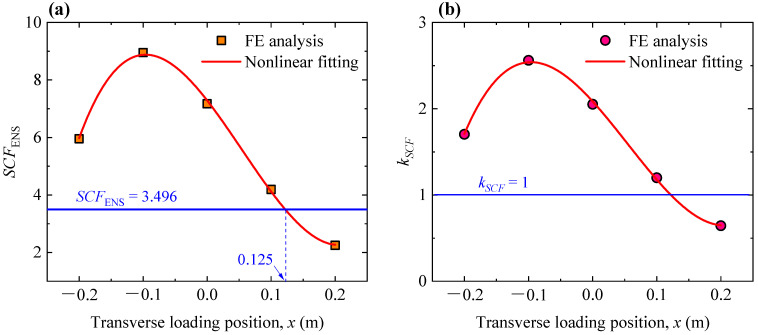
Effect of the transverse loading position on the ENS-based SCFs: (**a**) variation in the ENS-based SCFs; (**b**) variation in the amplification coefficient *k_SCF_*.

Both the effective notch stress (ENS) and nominal stress were obtained using the FE analysis; subsequently, the ENS-based SCF could be determined by the ratio of the ENS to the nominal stress. Based on the FE analysis, values of the ENS-based SCFs corresponding to different transverse loading positions were plotted in Figure 5a. Additionally, the SCFs predicted by Equation (1) was also plotted. Based on the geometric sizes of the rib–deck weld in FE model (i.e., *t_d_* = 14 mm, *t_r_* = 8 mm, 1 − *t_p_/t_r_* = 1.0, *l_w,d_* = *l_w,r_* = 6 mm, *θ* = 78°), the predicted value was calculated to be 3.496.

As can be seen from Figure 5a, the ENS-based SCF is considerably affected by the transverse loading position. The ENS-based SCF reaches the greatest value when the wheel load is applied at the position of *x* = −0.1 m. The cubic polynomial function was employed to fit the results with a final correlation coefficient (*R*^2^) of 0.997, as expressed by Equation (2).

(2) $${SCF}_{ENS}=7.28829-28.63279x-79.15843x^{2}+484.32571x^{3}$$

where *x* represents the position of the wheel loading, as shown in Figure 4.

It can also be seen that the ENS-based SCF is consistent with the value predicted by Equation (1) when *x* = 0.125 m. On this basis, it is feasible to modify Equation (1) by introducing the amplification coefficient (*k_SCF_*) to take into account the effect of the transverse loading position. The *k_SCF_* could be determined by Equation (3).

(3) $$k_{SCF}={SCF}_{ENS,x}/{SCF}_{ENS,x=0.125}$$

where *SCF*_ENS,_ *_x_* is the ENS-based SCF at arbitrary position of the wheel loading; *SCF*_ENS, 0.125_ is the ENS-based SCF corresponding to the position of *x* = 0.125 m.

Values of *k_SCF_* were plotted in Figure 5b. The cubic polynomial function was also employed to fit the results with a final correlation coefficient (*R*^2^) of 0.997, as expressed by Equation (4). Clearly, the ENS-based SCF corresponding to arbitrary weld geometry and transverse loading position could be determined by using Equations (1)–(4). Notably, the obtained results are only applicable to the OSDs without considering the asphalt surfacing.

(4) $$k_{SCF}=2.08475-8.19016x-22.64257x^{2}+138.53711x^{3}$$

### 4.2. Combined Effect of the Transverse Loading Position and Asphalt Surfacing

**Figure 6:**
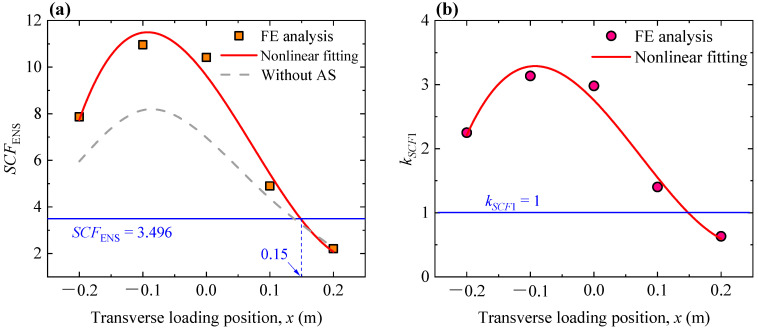
Combined effect of the transverse loading position and asphalt surfacing: (**a**) variation in the values of the ENS-based SCF, where AS represents the asphalt surfacing; (**b**) variation in the amplification coefficient *k_SCF_*_1_.

In practical engineering, the effect of asphalt surfacing is considerable. To investigate the combined effect of the transverse loading position and asphalt surfacing, FE models with a 60 mm thick asphalt surfacing were developed. The elastic modulus and Poisson’s ratio of the asphalt surfacing were assumed to be 5058 MPa and 0.3, without considering the variation in temperature. Values of the ENS-based SCF and *k_SCF_*_1_ were obtained, as plotted in Figure 6. The cubic polynomial function was also employed to fit the results, as expressed by Equations (5) and (6), and the final correlation coefficients (*R*^2^) were both 0.927. It could be seen that the ENS-based SCF considering the effect of asphalt surfacing is generally greater than the one without considering such effect. This further demonstrates that Equation (1) cannot be directly applied to practical engineering as the effect of asphalt surfacing is not involved. Notably, while considering the effect of the asphalt surfacing, there is no conflict between the increase in the ENS-based SCF and the decrease in the weld stress. The reason is that the ENS-based SCF is defined as the ratio of the ENS to the nominal stress, and both the ENS and nominal stress decrease when considering the effect of the asphalt surfacing but the decrease in the nominal stress is more obvious. For instance, when the wheel loading was applied at the position of *x* = 0.10 m, the ENS and nominal stress were 31.8 and 7.6 MPa without considering the effect of the asphalt surfacing, while the values decreased to be 25.0 and 5.1 MPa considering an such effect. It is obvious that both the ENS and nominal stress decrease, while the ENS-based SCF increases (increased from 4.18 to 4.90 in terms of this example) when considering the effect of the asphalt surfacing.

It can also be seen that the ENS-based SCF, considering the combined effect of the transverse loading position and asphalt surfacing, is consistent with the value predicted by Equation (1) when *x* = 0.15 m. On this basis, another amplification coefficient (*k_SCF_*_1_) was introduced to take into consideration the combined effect of the transverse loading position and asphalt surfacing, as expressed by Equation (7). On this basis, an ENS-based SCF which corresponds with arbitrary weld geometry and transverse loading positions, used in practical engineering, could be determined by using Equations (1) and (5)–(7).

(5) $${SCF}_{ENS}=9.63401-35.68153x-118.19119x^{2}+538.58958x^{3}$$

(6) $$k_{SCF1}=2.75572-10.20639x-33.80755x^{2}+154.05881x^{3}$$

(7) $$k_{SCF1}={SCF}_{ENS,x}/{SCF}_{ENS,x=0.15}$$

### 4.3. Effect of the Temperature of Asphalt Surfacing

**Figure 7:**
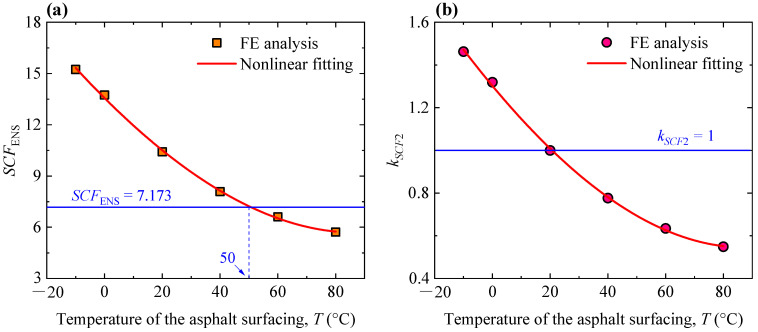
Effect of the temperature of the asphalt surfacing: (**a**) variation in the values of the ENS-based SCF; (**b**) variation in the amplification coefficient *k_SCF_*_2_.

In Section 4.2, the elastic modulus of the asphalt surfacing was valued as 5058 MPa, which corresponded to a temperature of 20 ℃ according to Equation (8). Additionally, the elastic modulus was set to be 16,290, 11,031, 2320, 1064, and 488 MPa to simulate the effect of temperatures of −10, 0, 40, 60, and 80 °C, respectively. The loading position remained unchanged (the wheel load was applied right above the U-rib, i.e., *x* = 0.0) to investigate the effect of the temperature of asphalt surfacing. Values of the ENS-based SCF in presence of different temperatures were determined by the FE analysis, as plotted in Figure 7a. It could be seen that the ENS-based SCF decreases with the increasing temperature of asphalt surfacing. Notably, the asphalt surfacing has a greater elastic modulus at lower temperatures, decreasing fatigue stress. At low temperatures, even though the rib–deck weld has a higher stress concentration, the stress range will be much smaller due to the strengthened composite stiffness of the asphalt surfacing and OSD. Furthermore, since the ENS-based SCF is sensitive to the transverse loading position (see Figure 6a), it is believed that the curve in Figure 7a changes with the variation in the transverse loading position. However, it is unnecessary to perform additional FE simulations to conduct the parametric analysis, since the predictive formulae for determining the ENS-based SCFs, corresponding to an arbitrary transverse loading position and the temperature of the asphalt surfacing, could be proposed based on the current database. If additional simulations can be performed to expand the database of the ENS-based SCFs, the only result would be that the relative error of the proposed formulae will decrease.

The cubic polynomial function was also employed to fit the results, as expressed by Equation (9), with a final correlation coefficient (*R*^2^) of 0.998. Taking the ENS-based SCF at 20 °C (i.e., *SCF*_ENS_ = 7.173) as the reference, another amplification coefficient (*k_SCF_*_2_) was introduced to determine the ENS-based SCF at arbitrary temperatures of the asphalt surfacing, as expressed by Equation (10). The values of *k_SCF_*_2_ are plotted in Figure 7b. The cubic polynomial function was also employed for nonlinear fitting, as expressed by Equation (11). The final correlation coefficient (*R*^2^) was 0.998. To this end, the ENS-based SCF corresponding to arbitrary weld geometry and temperatures in practical engineering could be determined by using Equations (1) and (8)–(11).

(8) $$E=11031\times 10^{-0.01693T}$$

where *E* is the elastic modulus of asphalt surfacing and *T* is the temperature.

(9) $${SCF}_{ENS}=13.56537-0.16886T+7.66876\times 10^{-4}T^{2}+1.52388\times 10^{-6}T^{3}$$

(10) $$k_{SCF2}={SCF}_{ENS,T}/{SCF}_{ENS,T=20}$$

(11) $$k_{SCF2}=1.30223-0.01621T+7.36178\times 10^{-5}T^{2}+1.46288\times 10^{-7}T^{3}$$

### 4.4. Validation of the Proposed Formulae

**Figure 8:**
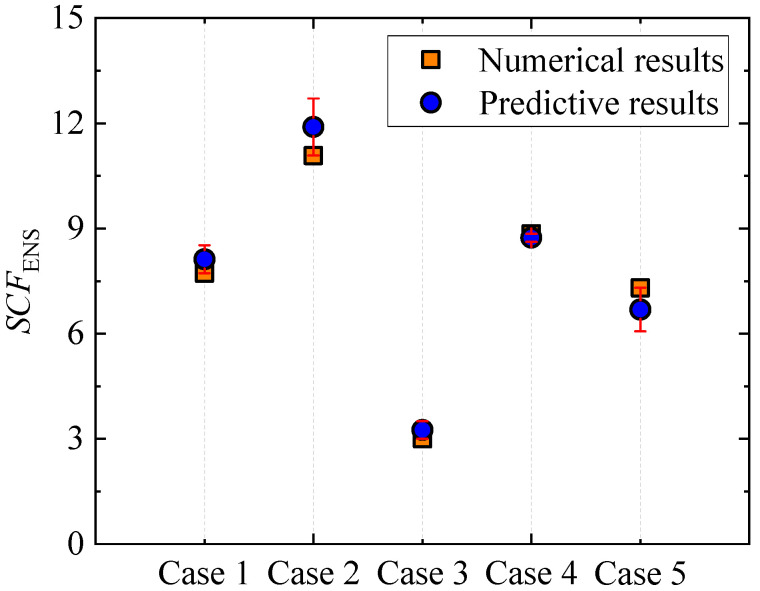
Comparison of the numerical and predictive results of *SCF*_ENS_.

To validate the feasibility and reliability of the proposed formulae, an additional five cases were considered, as listed in Table 1. Corresponding FE models were developed to obtain the FE results, and predictive results were also obtained by employing Equations (5)–(11). Both the numerical and predictive results were plotted in Figure 8 and Table 2. As can be seen, the ENS-based SCFs obtained using FE analysis were 7.73, 11.08, 3.01, 8.85, and 7.31, respectively, while those obtained by employing the proposed formulae were 8.12, 11.89, 3.25, 8.74, and 6.69, respectively. The relative errors between the numerical and predictive results were 5.14%, 7.34%, 8.36%, −1.27%, and −8.48%, which were all within 10%. Therefore, it is believed that the proposed formulae are acceptable for engineering applications. Additionally, to minimize the adverse effects of the relative error, the relative error should be reasonably considered so as to obtain the distributing range of the evaluating results of the fatigue damage.

## 5. Conclusions

The prediction of ENS-based SCFs of a rib–deck weld in an OSD, considering the effect of asphalt surfacing, was the focus of the present study. Based on the refined FE models, parametric analysis was conducted to investigate the effect of the transverse loading position, the combined effect of the transverse loading position and asphalt surfacing, and the effect of the temperature of asphalt surfacing. On this basis, predictive formulae for determining ENS-based SCFs for engineering applications were proposed by integrating the parametric equations without considering the effect of asphalt surfacing. The following conclusions can be drawn.

(1) ENS-based SCFs of rib–deck welds are sensitive to transverse loading positions, and the greatest value corresponds to the loading position of *x* = −0.1 m. The ENS-based SCF increases when considering the combined effect of the asphalt surfacing, while the variation trend remains unchanged.
(2) ENS-based SCFs are considerably affected by the temperature of asphalt surfacing. The lower the temperature is, the greater the ENS-based SCF is. And the ENS-based SCF decreases with an increase in temperature.
(3) Based on the numerical results of ENS-based SCFs, predictive formulae are proposed to determine the ENS-based SCFs corresponding to arbitrary weld geometry, transverse loading position, and the temperature of the asphalt surfacing. Validation research shows that the proposed formulae could provide reliable results with a relative error within 10% (namely 5.14%, 7.34%, 8.36%, −1.27%, and −8.48% for the five cases, respectively), which is believed to be acceptable for real-world engineering applications.

In future investigations, more cases considering variations in weld geometry, transverse loading position, and temperature should be simulated to extend the database of the ENS-based SCF so as to improve the predictive accuracy of the regressive formulae. Additionally, the artificial neural network (ANN) and machine leaning method should also be employed to propose more reliable models for the prediction of ENS-based SCFs.

## Figures and Tables

**Table 1:** Cases for validation.

Case	Transverse Loading Position (m)	Temperature of the Asphalt Surfacing (℃)
1	*x* = 0.05	*T* = 5
2	*x* = −0.05	*T* = 15
3	*x* = 0.15	*T* = 25
4	*x* = −0.15	*T* = 35
5	*x* = 0	*T* = 50

**Table 2:** Comparison of the numerical and predictive results.

Case	Numerical Results	Predictive Results	Relative Error (%)
1	7.73	8.12	5.14
2	11.08	11.89	7.34
3	3.01	3.25	8.36
4	8.85	8.74	−1.27
5	7.31	6.69	−8.48

## Data Availability

The data that support the findings of this study are available from the corresponding author upon reasonable request.
